# PB.16: Contrast-enhanced digital mammography: a single-centre experience

**DOI:** 10.1186/bcr3516

**Published:** 2013-11-08

**Authors:** SB O'Neill, DC O'Neill, N Marshall, L Duddy, MF Ryan, JE Barry

**Affiliations:** 1 Cork University Hospital, Cork, Ireland

## Introduction

Contrast-enhanced mammography is a relatively novel technique. We describe a single-centre experience with use of the technique to facilitate the detection and characterisation of lesions in the breast by triangulation of contrast-enhanced mammography, biopsy/gross pathological specimen and MR findings.

## Methods

Retrospective review of the pathological and imaging findings of consecutive patients undergoing contrast mammography was performed. Sixty-four contrast mammograms were performed on 61 patients (all female, mean age 54.3 ± 13.2 years) over a 15-month period. Technique involves a dual energy acquisition of CC and MLO views following the administration of contrast medium, then digital subtraction (GE Senographe DS).

## Results

At contrast-enhanced digital mammography, enhancement was observed in 37 of 43 patients with biopsy-proved cancers. Of the six patients with a cancer diagnosis but no enhancement, three were post neoadjuvant treatment. Three patients without a tissue diagnosis of malignancy demonstrated mammographic enhancement. Fourteen patients also underwent gadolinium-enhanced breast MR. Nine out of 10 patients with MR enhancement also demonstrated enhancement on contrast mammogram. One patient with no MRI enhancement had enhancement on contrast mammography. Morphology generally correlated with the pathologic diagnosis.

## Conclusion

The results of this study demonstrate the utility of contrast-enhanced mammography in the identification, evaluation and follow-up of breast lesions. It offers similar enhancement characteristics to MRI and represents a feasible alternative in centres without onsite MRI.

